# Immunoinflammatory Background of Neuronal Damage in Stroke

**DOI:** 10.3390/ijms24108619

**Published:** 2023-05-11

**Authors:** Antonino Tuttolomondo

**Affiliations:** Internal Medicine and Stroke Care Ward, Department of Health Promotion, Maternal and Infant Care, Internal Medicine and Medical Specialties “G. 6 D’Alessandro”, University of Palermo, Piazza delle Cliniche n.2, 90127 Palermo, Italy; bruno.tuttolomondo@unipa.it

Ischemic stroke is caused by a reduction in blood flow to the brain and is a major cause of mortality and disability worldwide. Cerebral ischemia initiates a complex cascade of events at genomic, molecular, and cellular levels, and inflammation is important in this cascade, both in the central nervous system (CNS) and the periphery [1,2,3]. Furthermore, recent research points to the role of chemokines, normally key factors in inflammation, in thrombo-genesis [3,4], probably also explaining the hyper-responsiveness of platelets in ischemic stroke. Recently, our group reported that lacunar strokes, compared to non-lacunar ones, exhibited significantly lower plasma levels of tumor necrosis factor (TNF)-α and interleukin (IL)1-β, P-selectin and intercellular adhesion molecule 1 (ICAM-1) 24–72 h (h) and 7–10 days after stroke onset [5]. One previous study examined inflammatory pathways in a lacunar study [6].

Inflammation is an important part of stroke pathophysiology, especially in the context of reperfusion. Several studies have shown that a nonspecific systemic inflammatory response occurs after both ischemic and hemorrhagic stroke, proving that inflammatory mechanisms intrinsic to the brain and the blood are among the important mediators of focal cerebral injury. The immune system is actively involved in the pathogenesis of acute brain damage through some events, such as leukocyte and monocyte infiltration into the brain, the activation of resident cells, including microglia, astrocytes, and endothelial cells, and several high serum levels.

Few studies have examined the relationship between inflammatory biomarker blood levels, cardioembolic stroke subtype, and neurological deficit. A study by our own [7] group examined levels of immuno-inflammatory variables in patients with cardio-embolic acute ischemic stroke compared to other diagnostic subtypes and to evaluate the relationship between immuno-inflammatory variables, acute neurological deficit, and brain infarct volume. One-hundred-twenty patients with acute ischemic stroke and 123 controls without a diagnosis of acute ischemic stroke were evaluated. Patients with ischaemic stroke classified as cardio-embolic (CEI) showed, compared to other subtypes, significantly higher median plasma levels of TNF-α, IL-6 and IL-1β. Furthermore, stroke patients classified as lacunar showed, compared to other subtypes, significantly lower median plasma levels of TNF-α, IL-6, and IL-1β. Multiple linear regression showed a significant association between the Scandinavian Stroke Scale (SSS) score at admission and diagnostic subtype, infarct volume of cardio-embolic strokes, and some inflammatory variables. Our findings confirm that cardio-embolic strokes have a worse clinical presentation and produce larger and more disabling strokes than other ischemic stroke subtypes, reporting a possible explanation of the higher immuno-inflammatory activation during the acute phase.

Animal models of focal ischemia induced by middle cerebral artery occlusion (MCAO) provide evidence for most of the cellular inflammatory responses in stroke.

In this Special Issue of the International of Molecular Sciences (IJMS), some authors reviewed the role of inflammatory cytokines in the pathogenesis of ischemic neuronal damage. We will also review the involvement of microglial cells, which are the major resident immune cells in CNS, and play a critical role in the immune response to a variety of events, including infection, trauma, stroke, and neurodegeneration. Among the various types of circulating leukocytes, neutrophils are generally the first leukocyte subtype recruited to the ischemic brain, and we will review their role in ischemic stroke pathogenesis. The inflammatory responses of the brain to post-ischemia are characterized by the involvement of different subtypes of T lymphocytes, which contribute to the pathogenesis of stroke-induced microvascular dysfunction and tissue damage. The authors also reviewed the role of T lymphocytes and their subsets. T-cells can be separated into three major groups based on function, as follows: cytotoxic T-cells (CD8+), helper T-cells (Th; CD4+), and regulatory T-cells (Tregs). We will review the literature on the available data concerning their involvement in ischemic stroke pathogenesis. We will also review the role of inflammatory mediators in a particular subtype of ischemic stroke, such as cardioembolic and atherosclerotic ones.

In the first article of this Special Issue, Carandina et al. [8] targeted the Autonomic Nervous System for Risk Stratification, Outcome Prediction and Neuromodulation in Ischemic Stroke.

In recent years, starting from the bidirectional relationship between autonomic nervous system (ANS) dysfunction and acute ischemic stroke (AIS), researchers have identified prognostic factors for risk stratification, the prognosis of mid-term outcomes, and the response to recanalization therapy. Impaired autonomic function, characterized by a predominance of sympathetic activity, is common in patients with acute ischemic stroke. Some of the variables used, such as autonomic activity markers [9], have been associated with worse outcomes, including cardiac complications, blood pressure variability changes, hyperglycemia, immune depression, disordered breathing while sleeping, thrombotic effects, and malignant edema. The involvement of the insular cortex has been suspected to play an important role in causing sympathovagal imbalance, but its exact role and that of other brain regions remain unclear. Although sympathetic overactivity in patients with ischemic stroke appears to be a negative prognostic factor, it remains to be seen whether therapeutic strategies that reduce sympathetic activity or increase parasympathetic activity might improve outcomes. In particular, the evaluation of ANS function through the analysis of heart rate variability (HRV) may appear to be a promising non-invasive and reliable tool for the management of patients with ischemic stroke [9]. Furthermore, preclinical molecular studies on the pathophysiological mechanisms underlying the onset and progression of stroke damage have shown an extensive overlap with the activity of the vagus nerve. Evidence from the application of vagus nerve stimulation (VNS) on animal models of AIS and patients with chronic ischemic stroke has highlighted the surprising therapeutic possibilities of neuromodulation. Preclinical molecular studies have highlighted that the neuroprotective action of VNS results from anti-inflammatory, antioxidant, and antiapoptotic mechanisms mediated by the α7 nicotinic acetylcholine receptor.

In the second article, Tuttolomondo et al. [10] reviewed the pathogenetic pathways involved in the molecular biology of atherosclerotic ischemic strokes. The etiopathogenetic burden of a cerebrovascular accident could be due to brain ischemia (~80%) or intracranial hemorrhage (~20%). The most common site wherein ischemia occurs is the site perfused by the middle cerebral arteries. Worse prognosis and disablement consequent to brain damage occur in elderly patients or those affected by neurological impairment, hypertension, dyslipidemia, and diabetes. After an ischemic stroke, diabetic patients experience an exorbitant activation of inflammatory molecular pathways, and ongoing inflammation is responsible for more severe brain injury and impairment, promoting the advancement of ischemic stroke and diabetes.

In the third article, Di Raimondo et al. reviewed the role of regular physical activity in neuroprotection against acute brain ischemia [11].

Neurotrophins such as brain-derived neurotrophic factor (BDNF), which is widely produced throughout the brain but also in distant tissues, such as muscles, has demonstrated regenerative properties and the potential to restore damaged neural tissue. Neurotrophins play a significant role in protecting and recovering function following neurological diseases such as ischemic stroke or traumatic brain injury. Unfortunately, the efficacy of the exogenous administration of these neurotrophins is limited by rapid degradation with subsequent poor half-life and a lack of blood–brain barrier permeability. Regular exercise seems to be a therapeutic approach that can induce the activation of several pathways related to the release of neurotrophins. Furthermore, exercise reduces the infarct volume in the ischemic brain and ameliorates motor function in animal models, increasing astrocyte proliferation, inducing angiogenesis, and reducing neuronal apoptosis and oxidative stress. One of the most critical issues is to identify the relationship between neurotrophins and myokines, newly discovered skeletal muscle-derived factors released during and after exercise, which can exert several biological functions. Various myokines (e.g., Insulin-Like Growth Factor 1, Irisin) have recently been shown to protect against neuronal injury in cerebral ischemia models, suggesting that these substances may influence the degree of neuronal damage in part via inhibiting inflammatory signaling pathways. Thus, the authors evaluated the main experimental data available to date on the neuroprotective and anti-ischemic role of regular exercise, also analyzing the possible role played by neurotrophins and myokines.

Further, Nikolic et al. [12] evaluated the genetic determinants that play an important role in the complex processes of inflammation and immune response in stroke and that could be studied in different ways. Inflammation and immunomodulation are associated with repair processes in ischemic stroke and, together with the concept of preconditioning, are promising modes of stroke treatment. One of the important aspects to be considered in the recovery of patients after a stroke is genetic predisposition, which has been studied extensively. Polymorphisms in a number of candidate genes, such as *IL-6*, *BDNF*, *COX2*, *CYPC19*, and *GPIIIa*, could be associated with stroke outcome and recovery. Recent GWAS studies pointed to the variant in genes *PATJ* and *LOC* as new genetic markers of long-term outcomes. The epigenetic regulation of immune response in stroke is also important, with mechanisms of histone modifications, DNA methylation, and the activity of non-coding RNAs. These complex processes change from the acute phase over the repair to establish homeostasis or provoke exaggerated reactions and death. The pharmacogenetics and pharmacogenomics of stroke cures might also be evaluated in the context of immuno-inflammation and brain plasticity. Potential novel genetic treatment modalities are challenged but they are still in the early phase of investigation.

Finally, Maida et al. [13,14] reviewed the neuroinflammatory mechanisms in ischemic stroke with a focus on cardioembolic stroke and speculated about future treatments. One of the most important causes of neurological morbidity and mortality in the world is ischemic stroke. It can be a result of multiple events, such as embolism of cardiac origin, the occlusion of small vessels in the brain, and atherosclerosis affecting cerebral circulation. Increasing evidence shows the intricate function played by the immune system in the pathophysiological variations that take place after cerebral ischemic injury. Following the ischemic cerebral harm, we can observe consequent neuroinflammation that causes additional damage provoking the death of the cells; on the other hand, it also plays a beneficial role in stimulating remedial action.

Immune mediators are the origin of signals with a proinflammatory position that can boost the cells in the brain and promote the penetration of numerous inflammatory cytotypes (various subtypes of T-cells, monocytes/macrophages, neutrophils, and different inflammatory cells) within the area affected by ischemia; this process is responsible for further ischemic damage of the brain. This inflammatory process seems to involve both the cerebral tissue and the whole organism in cardioembolic stroke, the stroke subtype that is associated with more severe brain damage and a consequent worse outcome (more disability and higher mortality) [15,16,17,18,19,20,21,22].
